# 532 Cross-Reactivity Between Topical and Systemic Sulfa Antibiotics

**DOI:** 10.1093/jbcr/irac012.161

**Published:** 2022-03-23

**Authors:** Ian A Powelson, Suvethavarshini Ketheeswaran, Charles S Hultman

**Affiliations:** LAC+USC Medical Center, Los Angeles, California; Johns Hopkins University School of Medicine, Baltimore, Maryland; Johns Hopkins University School of Medicine, Baltimore, Maryland

## Abstract

**Introduction:**

Silver sulfadiazine cream 1% is a sulfa derivative topical antimicrobial commonly used in burn care, in part due to its wide spectrum of activity, particularly against Pseudomonas aeruginosa. Contact dermatitis due to sulfadiazine component is a known but rare complication of silver sulfadiazine. In contrast, systemic sulfonamide antimicrobials are a commonly reported allergy and have been implicated in multiple hypersensitivity reactions. Some authors recommend avoidance of all sulfonamide antimicrobials regardless of route of administration for a patient with a known allergy to a sulfonamide antimicrobial due to structural similarities between all drugs in the class, resulting in a high risk of cross-reactivity. Clinical reports are lacking, however, and the few reports that do exist implicate silver as the culprit rather than the sulfadiazine component. Therefore, while the theoretical risk of reactivity to a topical application of sulfadiazine exists, there is no data to support this in practice.

**Methods:**

A retrospective review of 42 patients with a sulfa allergy who were admitted to the burn unit of an academic medical center between June 2016 and June 2021 were reviewed. Inclusion criteria were age > 18, burn requiring admission, sulfa allergy, and receiving topical sulfa antimicrobial therapy. Data on the reported allergen, reaction, and any adverse reaction from the use of topical sulfa agents were recorded.

**Results:**

Forty-two patients were identified. Of these, 32 (76%) reported a non-specific “sulfa” allergy, while 10 (24%) reported a specific allergy to trimethoprim-sulfamethoxazole. The reactions were reported as unknown (20, 48%), rash (9, 21%), hives (6, 14%), gastrointestinal (4, 9%), or other (3, 7%). One patient had a history of respiratory distress with trimethoprim-sulfamethoxazole administration. All 42 patients received treatment with topical silver sulfadiazine and 10 also were treated with topical mafenide acetate solution. There were no reported adverse events and no patients discontinued therapy. Two patients were identified with no previous sulfa allergy but who discontinued topical silver sulfadiazine due to burning sensation, a rare but previously reported side effect of this medication.

**Conclusions:**

Silver sulfadiazine remains one of the most commonly used topical antimicrobials in burn care due to its broad spectrum of activity, low cost, and ease of use. Adverse reactions to topical silver sulfadiazine and mafenide acetate are rare, even in those with reported sulfa allergies. Topical sulfa anti-microbials can likely be used safely in most patients with allergies to systemic sulfa antibiotics, and the rate of cross-reactivity appears to be low. A test patch is recommended prior to application over a large area.

